# SANDWICH GENERATION CAREGIVING: NEGATIVE RELATIONSHIP QUALITY AND BURNOUT

**DOI:** 10.1093/geroni/igae098.3138

**Published:** 2024-12-31

**Authors:** Sabine Lohmar, Erika Fenstermacher, Montgomery Owsiany, Catherine Ju, Barry Edelstein

**Affiliations:** West Virginia University, Morgantown, West Virginia, United States; West Virginia University, Morgantown, West Virginia, United States; West Virginia University, Morgantown, West Virginia, United States; West Virginia University, Morgantown, West Virginia, United States; West Virginia University, Morgantown, West Virginia, United States

## Abstract

The sandwich generation provides caregiving for both their children and their aging parents. These caregivers often experience low satisfaction, depression, and burnout, yet little is known about their contributing factors. For example, researchers have not investigated the role of relationship quality among sandwich generation caregivers, who face unique challenges. This study is the first to investigate relationship quality as a moderator between informal caregiver burnout and depression. Regression analyses included measures of positive and negative relationship quality and informal caregiver burnout as predictors and depressive symptoms as the criterion. Correlational analyses indicated that positive relationship quality (r = -.414, p <.001; r = -.274, p =.005) and negative relationship quality (r =.721, p <.001; r =.467, p <.001) were significantly associated with informal caregiver burnout and depression, respectively. Positive relationship quality significantly moderated the association between caregiver burnout and depression for sandwich generation caregivers only (R² =.323, F[3, 99] = 15.741, p <.001, β =.019, p =.006), as did negative relationship quality (R² =.307, F[3, 96] = 14.184, p <.001, β = -.016, p =.046). Among sandwich caregivers, positive relationship quality buffered the relation between caregiver burnout and depression, while negative relationship quality enhanced this relation. Positive and negative relationship quality were not significant moderators among individuals who care for parents only or children only. These results suggest that, for sandwich generation caregivers, relationship quality is an important contributing factor for caregiver burnout and depression.

